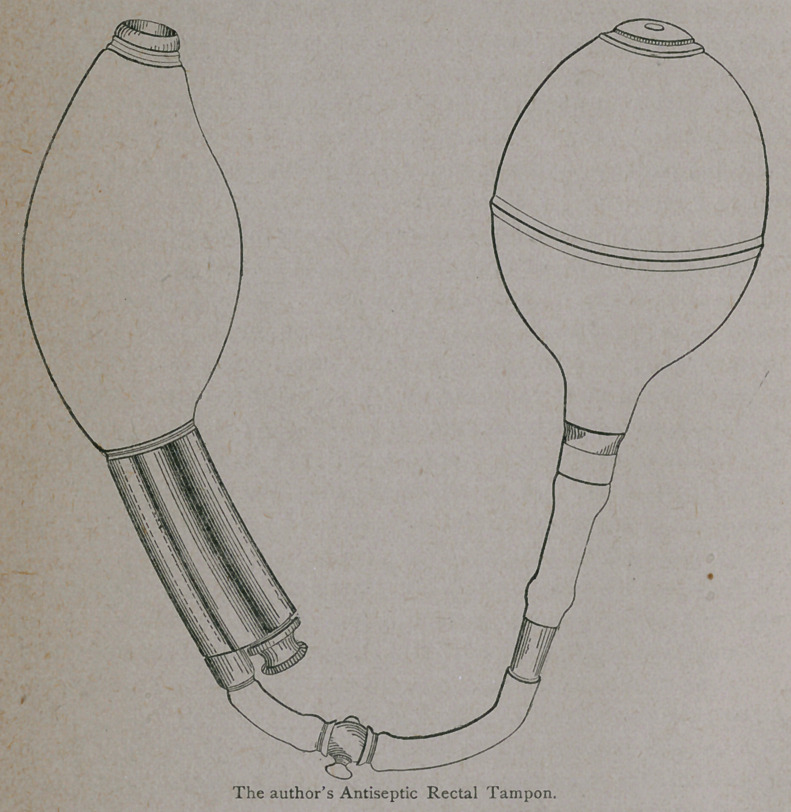# Hemorrhoids—Their Diagnosis and Treatment

**Published:** 1890-02

**Authors:** Edward Clark

**Affiliations:** Buffalo, N. Y.; 271 Franklin Street


### HEMORRHOIDS—THEIR DIAGNOSIS AND TREATMENT.

By EDWARD CLARK, M. D., Buffalo, N. Y.

Since I have been engaged in the practice of rectal surgery as a specialty, I have received a number of requests from some of my professional confrères, to prepare, for publication, an article on the subject which forms the title of this paper. In complying with this request, it will be my aim to group the salients of this subject for convenient reference by those who feel interested therein—to present a *résumé* of the most approved methods adopted by rectal surgeons in the management of this malady—rather than to set up original or new claims for special methods of dealing with it.

I am convinced, both by observation and experience, that the profession generally is possessed of only a vague and indefinite knowledge of the proper management of this very common and distressing affection. Absence of special instruction and want of opportunity to study this class of diseases, with a lack of the proper facilities for diagnosis and treatment, have led to this want of familiarity with the subject on the part of the profession.

Rectal disorders are, perhaps, as common as any class of diseases to which flesh is heir, and they are productive of a vast amount of suffering and misery, as the rectum has to do with one of the most important functions in the human economy. A degree of nervous depression and an anxiety of mind are frequently met with in patients suffering from rectal troubles, which is truly astonishing and out of all proportion to the severity of the disorder itself. To many physicians, rectal diseases seem so repulsive and unattractive, that their pathology and treatment have not received that degree of thoughtful consideration to which their importance entitles them. This apathetic attitude of a majority of the profession toward this important field of study has been the most powerful agency in relegating the treatment of these distressing maladies to the domain of quackery. Patients suffering from rectal diseases apply to their family physician for relief, and after becoming discouraged and disgusted with the *attempted methods* of treatment resorted to, at last become the prey of ignorant pretenders, whose flaming advertisements so easily captivate the minds of those who suffer from chronic ailments.

I have used the term "attempted methods" of treatment, because both observation and experience lead me to believe that aside from those practitioners who are regularly engaged in a surgical practice, there are very few physicians, indeed, who ever attempt to do anything in the way of a radical cure for hemorrhoids. They simply content themselves with making a few futile efforts at mitigating the sufferings of their patients with piles, by the use of ointments, laxatives, enemata, and the like. This indifference, on the part of the profession, relative to the treatment of piles, has been the means of infusing into the minds of many of the laity the erroneous conviction that the hemorrhoidal disease is an affection presenting but slight prospects for alleviation, and, acting under the belief that "what cannot be cured must be endured," they drag out a wretched existence, suffering more or less from an affliction which of all maladies is, perhaps, most readily amenable to proper treatment. I verily believe that there are physicians who labor under the delusion that hemorrhoids are incurable, as witnessed by the remark of one, who, when consulted by a patient with a bad case of piles, told him that if he could cure that disease he would be able to ride in a chariot of gold.

Success in the treatment of rectal disease, more especially hemorrhoids, rests very largely upon the physician's ability to make a correct diagnosis in each and every case presenting itself to him for treatment. In the majority of cases this cannot be done without a most thorough and careful examination. Specula of various kinds may be used, and one familiar with their proper use may learn much by this method of examination; but to the inexperienced, the results of specular examination, as a rule, are very unsatisfactory. Digital examination is generally fruitful of much valuable information. The only way, however, to make a thoroughly satisfactory examination of the rectum, is to stretch and temporarily paralyze the sphincter ani muscle, in order that at least the lower three or four inches of the rectal mucous membrane may be brought into view and carefully examined. The surgeon who always pronounces a positive opinion on a rectal disorder, without such examination, will sooner or later fall into grave errors which may injure his reputation not a little. In order to make this examination properly, it is necessary to resort to the use of an anesthetic; to stretch the sphincter muscle, as has been done, without anesthetizing the patient, is a barbarous and cruel procedure which cannot be too strongly condemned. A great many patients object seriously to taking an anesthetic for diagnostic purposes; but if they are informed that any operation which may be necessary, can be done at the same sitting, we will, in the majority of cases, perhaps, be able to overcome their objections to the anesthesia.

Figure 1.

For office examinations, a firm examining chair, or table of some kind, is necessary. The number of these now offered to the profession is legion. Some of them reflect great credit on the skill and ingenuity of their inventors, but many others, in my opinion, are too complicated to be of much practical utility. One of the best that I have seen,

Figure 2.

and the one I use in my office, is that invented and manufactured by Dr. B. H. Daggett, of this city, known as the "Daggett operating and examining table," which, when not in use, makes a very convenient and ornamental office table.

Figure one shows the table when ready for use, and figure two shows it opened and extended for general anesthesia, or any surgical examination.

I am in the habit of directing my patients to take a brisk cathartic on the evening preceding the expected examination, and on the following morning after the bowels have been emptied, to wash out the rectum two or three times with warm water. This cleanses the bowel thoroughly, and nothing is left to interfere with the examination.

Having thus prepared the patient, we now proceed to make our diagnosis. It may seem to some that to diagnosticate hemorrhoidal troubles is essentially an easy matter. This is true, so far as the expert is concerned, but it certainly is not true as regards the average general practitioner, many of whom take a patient's word for it, that he or she has piles. During the past two years I have had a number of patients sent to me by reputable and highly intelligent physicians to be treated for hemorrhoids, when a careful examination would reveal the fact that they were suffering from some affection of the rectum other than such disease. One case was sent as hemorrhoids, which proved to be a fissure of the rectum; another patient who was supposed to have hemorrhoids, was suffering from papillomatous growths in the rectum; another had rectal polypi; and still another chronic ulceration with condylomatous growths around the anus. I cite these few cases, simply to show that a correct diagnosis in cases of rectal disease is not always made by physicians. We meet with many cases of pruritus ani, which are styled by patients and by many physicians as itching piles. My experience is that in these cases the itching and burning, which are so distressing to the unfortunate patient, are generally secondary to some trouble inside the rectum, as ulceration or some other disorder, upon the cure of which the itching and burning speedily disappear.

Kelsey defines hemorrhoids as "varicosities of the anal or rectal vessels." Hence, an external hemorrhoid is an affection of the subcutaneous vessels of the ano-rectal region, and is situated below the sphincter ani muscle. It consists, generally, of an enlargement, or rupture of a vein, outside of the anus. When the vein ruptures or breaks, a clot of blood of greater or less size is formed in the subcutaneous cellular tissue. This form of hemorrhoid, generally, comes on quite suddenly, and forms a tense, painful, bluish tumor, situated just at the verge of the anus. When the external hemorrhoid is due to a saccular dilation of a vein, its growth is more gradual, and is always, of course, aggravated by straining at stool. If left to themselves, either of these forms of growth undergoes changes. The clot may become absorbed and the growth disappear; or the clot may remain for a greater or less period of time; the tumor then becomes hardened, less painful, and finally results in tags or tabs of skin which are also known as a variety of external hemorrhoids. An external hemorrhoid, when first formed, is extremely painful and annoying, causing a degree of suffering and uneasiness out of all proportion to the magnitude of the affection. If not properly treated at this time, it sometimes becomes greatly inflamed, and may result in suppuration. The treatment of this variety of hemorrhoids is quite simple, and followed by almost immediate relief from suffering. All that is necessary is to incise the tumor in its longitudinal axis and turn out the clot completely. This little operation, formerly so painful, may be done almost painlessly, by first drawing along the line of incision a wooden toothpick dipped in pure carbolic acid. After the cavity is thoroughly emptied of its contents, a shred of lint may be loosely placed in it to prevent primary union, and the patient instructed to wear a wad of absorbent cotton, and, perhaps, a bandage for a few days, to prevent soiling his linen.

External hemorrhoids are always venous, but an internal hemorrhoid may be venous, arterial, or capillary.

External hemorrhoids, in many cases, do not drive the patient to seek surgical advice, but patients suffering from internal hemorrhoids, are sooner or later—generally later—compelled to seek professional advice, owing to the train of morbid phenomena that always follows in the track of internal hemorrhoids.

Internal hemorrhoids always tend to grow worse if left without treatment, and it is unfortunate that a sense of false modesty keeps many patients, especially women, from consulting a physician for this very common and distressing, but easily cured, malady. As a genuine fact, it may be stated that an internal venous hemorrhoid is an affection of the internal hemorrhoidal veins. It will be remembered that these veins pass upward beneath the mucous lining of the rectum, and pass through the muscular coat, through little "button-hole" like openings; they then unite with other venous trunks and help to form the portal vein. The superior hemorrhoidal veins have their origin in little blood sacs, which lie beneath the mucous membrane, just above the anus. Each one of these little sacs is connected by a small anastomatic venous twig with the external hemorrhoidal veins, thus establishing a direct communication between the portal and general venous systems. Some writers hold that these little blood sacs are incipient hemorrhoids, that enlarge principally by the contraction of the muscular fibers surrounding the button-hole like foramina through which the internal hemorrhoidal veins pass from the rectum. I am inclined to believe, however, that these little blood sacs, or venous spaces, are normal anatomical structures. I am also inclined to the belief that pressure on the veins from various causes, such as constipation, straining in defecation, and portal obstruction from whatever cause, is more important as an etiological factor in internal hemorrhoids, than the contraction of the so-called "button-holes" in the muscular coat of the rectum.

Internal hemorrhoids are liable to be confounded with prolapse of the mucous coat of the rectum,and with polypoid growths and papillomata. In prolapsus, the portion extruded from the anus, generally, completely encircles the anal aperture, and has, unless permanently outside of the body, the bright red color and appearance of the normal mucous membrane. When an internal hemorrhoid of the venous variety is pressed below the sphincter ani muscle, it appears as a tense rounded or oval-shaped tumor of a bluish red or purple color. When some degree of prolapse accompanies it, there will be seen a distinct furrow of separation between it and the hemorrhoid, and the contrast between the colors of the protruding masses is very striking indeed. If the hemorrhoid partakes of the arterial character, it is generally somewhat lighter in color, and when strongly compressed by the sphincter muscle, a small jet of arterial blood is not unfrequently seen to issue from it *per saltem*. The capillary, or, perhaps more properly, the arterio-capillary hemorrhoid, is the form which, in my experience, is attended with the greatest amount of hemorrhage. This variety is rarely ever seen outside of the rectum, and consists of an elevated, roughened, aggregation of small capillary and arterial twigs under the rectal mucous membrane. The surface of this growth becomes, by friction and pressure, broken and evoked, and from which, especially after defecation, there escapes quite a copious discharge of blood. This growth properly constitutes the so-called "bleeding piles," about which we hear so much from the laity.

### TREATMENT.

I shall add nothing to what I have already said on the treatment of external hemorrhoids.

The treatment of internal hemorrhoids may be discussed under the two heads of operative, and non-operative, or palliative.

The non-operative or palliative measures are, perhaps, most resorted to by the ordinary practitioner. They do not, in severe cases, very often effect a cure, but in recent cases, before structural changes of any great extent are brought about, they are, if persistently and faithfully carried out, productive of much good, and greatly relieve the sufferings of the patients afflicted. Astringent washes, ointments, laxatives, enemata, hot sitz baths, and suppositories all have their place and use as palliative measures. Every physician is familiar with the therapeutic uses and mode of action of the above-named agents, and it would be out of place and a waste of time for me to discuss them in detail at this point.

One thing, however, that must be cautiously guarded against, in the palliative treatment of hemorrhoids, is constipation. If this condition is allowed to prevail, all efforts at palliation of the trouble will be in vain. This condition acts as a great evil in hemorrhoidal troubles, by compressing the hemorrhoidal veins, and it should be obviated and removed by such of a combination of medicine, exercise, and regulated diet, as will secure, at least, one soft unirritating passage of the bowels daily.

Among the astringents, which may be useful as palliative measures, we find tannin, alum, zinc sulphate, acetate of lead, carbolic acid, and extract of hammamelis. These different agents are used in various combinations, and of different strengths according to the indications to be met in individual cases. Among the drugs, which are useful in suppositories, we may mention opium, cocaine, belladonna, iodoform, and bismuth. The two last named, combined with cocaine, have given me much satisfaction. It should be borne in mind, that washes containing any drug should not, as a general rule, be more than one-third as strong as ointments containing the same substance.

The object to be attained by the use of any astringent wash or ointment, is to get a mild astringent effect, instead of an irritation which will produce expulsive efforts on the part of the rectum.

If the above means are intelligently applied, and the patients kept for some time in the horizontal position, much may be done to mitigate the sufferings of those patients who cannot, or will not, submit themselves to the operative plan of treatment.

Various operative procedures have been resorted to for the radical cure of internal hemorrhoids, such as stretching the sphincter ani muscle, ligature, injection of various substances into the pile, clamp and cautery, crushing, ecraseur, and excision.

Perhaps the simplest of all these methods is that of gradual dilatation of the anal sphincters. The most noteworthy advocates of this method are the distinguished Frenchmen, Prof. Verneuil and M. Fontan. It is claimed by these authorities and others, that the great majority of all cases of internal hemorrhoids can be cured by this method. In resorting to this manipulation, it is necessary to use an anesthetic, as the dilatation must be complete and thorough, and to accomplish this without an anesthetic is a very painful manipulation, indeed. American surgeons are not so enthusiastic in their praise of this method as the French surgeons. This method is, perhaps, most applicable to the treatment of recent cases, and, perhaps, should receive a trial in those cases where the patients have a horror of the knife, ligature, or cautery.

The treatment of hemorrhoids by ligature is very ancient, indeed. Hippocrates and Celsus both speak of it, and describe how "the operation" should be carried out. Allingham, of London, is, perhaps, the most noted advocate of this method of treatment. Many British and Continental surgeons, as well as many of our best American surgeons, also advocate the ligature for the cure of piles. It undoubtedly has many commendable advantages. It is easily applied, and generally followed by satisfactory results. Death after this operation is very rare, indeed. Of 5,863 patients, operated on by Allingham, only six died, or about one in a thousand. I do not think, however, that so far as mortality is concerned, this method of operation is any more safe than operation by excision (Whitehead's method) or the clamp and cautery. While it has its advantages, it also has its drawbacks. My experience is that the suffering of the patient, after the operation by ligature, is infinitely more severe than after the clamp and cautery operation, and the cure is certainly not more complete. I am partial to the clamp and cautery operation, because it is not difficult to perform ; it is followed by perfect results when properly executed, and, as I have said, the sufferings of the patient are not at all severe after the operation. This last advantage is worthy of careful consideration, especially in the case of a sensitive, nervous patient.

Another advantage which the clamp and cautery operation has over that by the ligature, is that it is liable to be followed by much less disturbance of the bladder and consequent use of the catheter.

Some writers claim that dangerous concealed hemorrhage frequently follows the operation by clamp and cautery. I hardly believe this can be true, if the operation is properly performed. When it does occur, it is either due to carelessness on the part of the operator, or it occurs in patients of a hemorrhagic diathesis.

If I have any suspicion that a patient on whom I operate is a bleeder, I make use of an instrument which I devised some time ago, and which is made for me by Tiemann & Co., of New York. I have called it the "antiseptic rectal tampon." It is delineated in the cut, a glance at which reveals its mode of action. The dilating bulb consists of soft rubber, attached to the hard rubber pipe, which passes through it. The hard rubber pipe is hollowed out its entire length, so that while remaining in the rectum flatus may escape, and, if necessary, the rectum may be washed out without removing the tampon or lessening the pressure on the dilating bulb. It is introduced into the rectum in a collapsed condition, and filled by means of the hand-bulb. When distended to the requisite degree, the small stop-cock is turned and the hand-bulb detached. The air enters the dilating bulb through the small opening of the tube which runs parallel to its large central channel, its inner extremity opening at the side of the large tube inside of the dilating bulb. As the vessels from which the bleed-

The author's Antiseptic Rectal Tampon.

ing occurs come from above downwards, the tampon acts by compressing them above the seat of operation. If any bleeding occurs, it is sure to show externally, as the pressure of the bulb prevents the blood from passing upward into the bowel. It is checked, of course, by increasing the pressure on the dilating bulb. Instead of air, ice water or hot water, may be used to inflate the bulb. This tampon may be used to check hemorrhage from the rectum after any of the operations for hemorrhoids, if it should occur, instead of the method of packing with sponge and lint, the insertion and removal of which is dreaded by patients more than the original operation.

As my experience with the operation by crushing and excision has been somewhat limited, I shall say nothing about these methods, except that I am very favorably impressed by the Whitehead operation, for the reason that it seems to me to possess many strong points as a scientific surgical procedure. The principal objection to it, so far as I can see, is that when it fails to produce primary union, there is some liability to the formation of anal stricture, owing to the contraction produced by the healing of the wound, which completely encircles the anus.

It is an operation which requires considerable skill and dexterity for its proper performance, and it sometimes is attended with considerable hemorrhage. I shall conclude this article with a few remarks on a method of treating hemorrhoids, which has been highly extolled by some, and vigorously condemned by others; a method which has had claimed for it wonderful results, which claim prolonged experience and careful observation, have shown to be almost entirely without foundation; a method which is now largely practised by the ignorant and illiterate on all cases of hemorrhoids coming under their observation. I refer to the process of injecting hemorrhoids with various substances, chief of which is a combination of carbolic acid with olive oil, or glycerine. That this method has no definite value as a curative agent I do not attempt to affirm, but that it has accomplished all that is claimed for it, and that it is applicable to the treatment of each and every variety of internal hemorrhoid, I do most emphatically deny. While I admit that it is useful in the treatment of a few selected cases, I am certain that the indiscriminate injection of carbolic acid into all varieties of hemorrhoids by persons who do not thoroughly understand the anatomy and treatment of these cases, is a practice which is productive of dangerous results, and one which cannot be too strongly condemned.

Many writers have attributed the discovery of this mode of treatment to the quacks, but it no doubt was first practised by a young physician of Illinois nearly twenty years ago. It was found to produce some very good results in a few cases, and he conceived the idea of selling the right to practise his secret mode of treatment to anyone who was willing to pay him for the privilege. The persons buying this privilege, many of whom were impecunious and unscrupulous physicians, were obliged to purchase from him also all of the solutions which they used in their practice. The western portions of our country, particularly, were soon flooded with the itinerant or traveling

"pile doctors," who went up and down seeking those who, for a consideration of some magnitude, wished to be cured of piles by a painless and harmless (?) method. Their armamentarium chirurgicum consisted of a bottle of carbolic acid solution and a hypodermic syringe. The wonderful tales told of the cures they made, with nothing said of their many failures, induced hundreds to go into this business, so that fortunes were made by those who sold the right to practise the secret system to each other. The medical profession got hold of the secret and lost no time in testing its merits. Many of the best men in the profession were carried away with it, and were as sanguine in their predictions of what it could accomplish as were the quacks themselves. Twenty years of experience and observation, however, have convinced many that this method of treatment has only a very limited field of usefulness, and a careful collection of statistics shows that it has been productive of much harm. In "Andrews' Rectal and Anal Surgery," a work recently published in Chicago, we find the following: "It is the old experience over again. Twenty years ago the profession was charmed by the results of coagulating injections thrown into venous enlargements in other parts of the body, but we were soon stopped by the occurrence of deaths from embolism. The hypodermic injection of piles confronts us with similar dangers.

"The following accidents have been reported out of about 3,304 cases: Deaths, 13; embolism of liver, 8; sudden and dangerous prostration, 1; abscess of liver, 1; dangerous hemorrhage, 10; permanent impotence, 1; stricture of rectum, 2; violent pain, 83; carbolic acid poisoning, 1; failed to cure, 19; severe inflammation, 10; sloughing and other accidents, 35."

Compare this with the records of the same number of cases of operation by the clamp and cautery, or ligature!

Kelsey, who first wrote favorably of this method, now says that he applies the plan mainly to selected cases of completely internal piles of moderate size, and having well-defined pedicles. In the book from which I have made the above quotation (Andrews), we read: "We were long ago reluctantly compelled to admit that these injections are dangerous, and until some way of avoiding the perils is shown we cannot recommend them except in special and selected cases."

The "Brinkerhoff System" of treating piles is nothing more or less than the carbolic acid treatment, and the formula for the celebrated secret pile remedy of this "system" is as follows:

Carbolic acid, one ounce.
Olive oil, five ounces.
Chloride of zinc, eight grains.

In using injections for piles there is a great danger of clots or globules of the injection being carried to the liver or heart. To avoid this some authors have recommended plugging of the rectum for twenty-four hours after the operation.

My rectal tampon, which I have already described, might be useful for the same purpose. It is also useful in these cases when introduced into the rectum and distended. By making downward traction with it, it not only distends the piles, but helps to turn them out of the rectum and keeps them in a position easy for treatment.

In concluding, let me quote again a paragraph from Andrews, which exactly expresses my own views on this subject: "Up to the present time science has not discovered any method of wholly avoiding the risks of the hypodermic injections. The method is moderately, but positively, dangerous, and we cannot recommend it as proper in ordinary cases."

271 FRANKLIN STREET.

## Figures and Tables

**Figure 1. f1:**
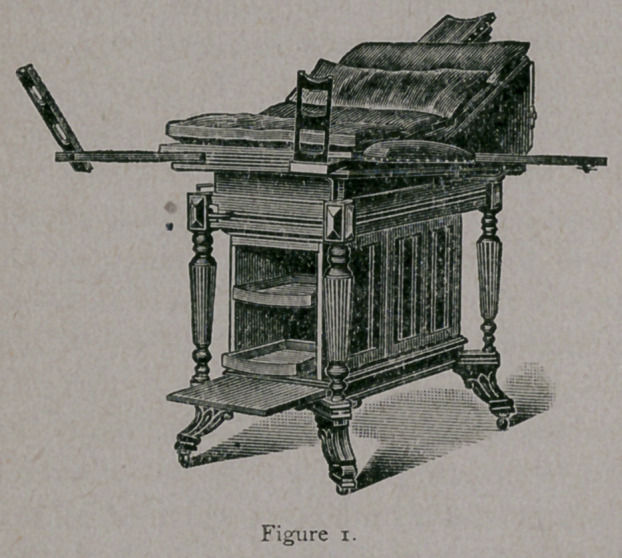


**Figure 2. f2:**
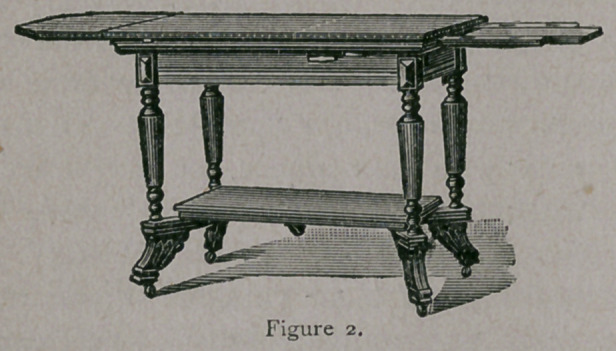


**Figure f3:**